# Ventral Visual Pathway-Cerebellar Circuit Deficits in Alcohol Dependence: Long- and Short-Range Functional Connectivity Density Study

**DOI:** 10.3389/fneur.2019.00098

**Published:** 2019-02-12

**Authors:** Lingling Chen, Bi-Xia Liu, Run Liu, Jiyong Zheng, Xi-Jian Dai

**Affiliations:** ^1^Department of Pediatric Internal Medicine, Linyi Central Hospital, Linyi, China; ^2^Department of ICU, Jiangxi Provincial Cancer Hospital, Nanchang, China; ^3^Department of Radiology, The Affiliated Xi'an Central Hospital of Xi'an Jiaotong University, Xi'an, China; ^4^Department of Medical Imaging, The Affiliated Huaian No.1 People's Hospital of Nanjing Medical University, Huai'an, China; ^5^Department of Medical Imaging, Jinling Hospital, Medical School of Nanjing University, Nanjing, China

**Keywords:** alcohol dependence, functional connectivity density, visual pathway, cerebellar circuit, receiver operating characteristic

## Abstract

**Objective:** To identify the underlying intrinsic functional connectome changes in patients with alcohol dependence.

**Methods:** A functional connectivity density (FCD) analysis was used to report on the functional connectivity changes in 24 male patients with alcohol dependence (age, 47.83 ± 6.93 years) and 24 healthy male subjects (age, 47.67 ± 6.99 years). We defined the voxels with a correlated threshold of *r* > 0.25 inside their neighborhood (radius sphere ≤ 6 mm) as shortFCD, and radius sphere > 6 mm as longFCD. We repeated the network analysis using a range of correlation r thresholds (*r* = 0.30, 0.35, 0.40, 0.45, 0.50, 0.6, and 0.75) to determine whether between-group differences were substantially affected by the selection of the different R-value thresholds used. A ROC curve was used to test the ability of the FCD in discriminating between the two groups. Pearson's correlation was used to evaluate the relationships between the FCD differences in brain areas and demographic characteristics.

**Results:** The covered differences in brain areas in binarized shortFCD were larger than binarized longFCD in both groups. The intra-group FCD differences did not depend on the selection of different thresholds used. Patients with alcohol dependence were associated with the longFCD deficit in the cerebellum posterior lobe, and shortFCD deficit in the ventral system of the visual pathway and increased shortFCD in the left precentral gyrus, right salience network and right cingulate gyrus. A ROC curve demonstrated that these specific brain areas alone discriminated between the two groups with a high degree of sensitivity and specificity. In the alcohol dependence group, the cerebellum posterior lobe, visual association cortex and the salience network displayed significant correlations with demographic characteristics.

**Conclusions:** The shortFCD analysis was more sensitive than the longFCD analysis in finding differences in the brain areas. The ventral visual pathway-cerebellar circuit deficit appeared to be altered in patients with alcohol dependence.

## Introduction

Alcohol dependence is the widest substance addiction worldwide, with high morbidity or mortality. Alcohol dependence is a chronic relapsing disorder, characterized by morbid alcohol consumption. Alcohol consumption creates a perception of relief from the negative emotions, but extravagant alcohol consumption may bring numerous adverse health consequences, such as cancer, liver cirrhosis and vehicle accidents (1). However, it may also thereby increase and/or reinforce the likelihood of future drinking behavior (2).

The pernicious effects of alcohol dependence on the brain and behavior are well-recognized. Neuroimaging studies have described diverse pernicious effects from alcohol dependence, including the neurochemical changes and regional functional activity in the brain (3). Resting-state functional MRI (rs-fMRI) can be used to visualize the brain activities associated with oxygenation and blood flow changes, without the need of exposure to radioactive tracers, which is widely used for detection of specific regional brain alterations that can't be identified by traditional MRI examination (4). The advances of rfMRI may help us to non-invasively explore the functional organization in the human brain, thus better characterizing the changes of regional neuronal spontaneous brain activity and intrinsic connectivity patterns, to better understand the underlying neural basis of neuropsychiatric disorders.

Functional connectivity studies have revealed abnormal connectivity patterns in individuals with alcohol dependence (5, 6). Seed-based functional connectivity analysis reflects the relationships of time series between a given seed point area and other brain areas (6, 7); however, it is based on a *priori* hypothesis with the need of a *priori* definition for the regions of interest. Functional connectivity density (FCD) can be used to identify the distribution of the brain network hubs and unbiasedly search for abnormalities within the whole brain, without the need of a *priori* hypothesis (7, 8). The FCD can be divided into short-range FCD (shortFCD) and long-range FCD (longFCD) on the basis of the neighboring relationships between brain voxels (9).

Alcohol dependence is associated with changes in regional brain activity in several areas, which makes its neurobiological mechanism more complex. Although recent evidence of structural and functional MRI experiments have increased tremendously, identifying several brain regions that are relevant to alcoholism (10, 11), the neurobiological mechanism underlying alcohol dependence remains largely unknown. The shortFCD and longFCD analysis can reveal extra information which cannot be provided by the seed-based functional connectivity analysis and FCD, and have been widely applied into several diseases (12–16). In the current study, we hypothesized that alcohol dependence is associated with distinct connectivity patterns reflected by different shortFCD and longFCD changes, depending on the neighborhood strategy. To test the hypothesis, we utilized the potential biological indicators of shortFCD and longFCD to characterize the intrinsic functional connectivity changes in 24 patients with alcohol dependence relative to 24 status-matched healthy subjects. Next, we used Pearson's correlation to evaluate the relationships between those brain areas with FCD differences and behavioral characteristics. We also utilized the receiver operating characteristic (ROC) curve to investigate whether the brain areas with FCD differences had the ability to distinguish between patients with alcohol dependence, and the status-matched healthy subjects.

## Materials and Methods

### Subjects

A total of 24 male patients with alcohol dependence (age, 47.83 ± 6.93 years; education, 9.67 ± 3.09; mean ± standard deviation) and 24 male healthy subjects (age, 47.67 ± 6.99 years; education, 8.33 ± 3.21 years) participated in the study.

Patients with alcohol dependence met the diagnostic criteria as defined by the Diagnostic and Statistical Manual of Mental Disorders, version 4 (DSM-IV). All recruited volunteers met the following inclusion criteria as in previous studies (6): (1) first-time visitors, and previously had never taken any benzodiazepine or chlormethiazole medications treatment before; (2) without any history of other substance dependence or abuse (such as marihuana and tobacco addiction) as defined by DSM-IV; (3) without any history of sleep disorders and major psychiatric disorders; (4) right-handedness; (5) without any family history of alcohol dependence. Additional exclusion criteria were as follows: (1) a history of head injury with loss of consciousness; (2) the presence of any past or current neurological disease; (3) presence of hepatitis C, hypertension and type 2 diabetes that required medical intervention; (4) aged above 60 years and below 19 years; (5) the presence of any contraindications to an MRI; and (6) any foreign implants, pathological brain MRI findings, and inborn or other acquired diseases. The life history of psychiatric disorders, daily alcohol consumption, severity of alcohol dependence questionnaire (SADQ), mean years of drinking and alcohol use disorders identification test (AUDIT) were recorded by an experienced psychiatrist who worked for more than 10 years. The study was approved by the Medical Research Ethical Committee, in accordance with the Declaration of Helsinki. Written informed consent was obtained from all volunteers.

### MRI Parameters

We performed the MRI scan on a 3.0-Tesla MR scanner (Siemens). First, 176 high-resolution anatomical images of 3D T1-weighted in an sagittal orientation (repetition time/ echo time (TR/TE) = 1,950/2.3 ms, gap/thickness = 0/1 mm, field of view (FOV) = 244 × 252 mm, acquisition matrix = 248 × 256, flip angle = 9°) were collected. Next, we collected 240 functional images (TR/TE = 3,000/25 ms, gap/thickness = 0.5/5.0 mm, flip angle = 90°, acquisition matrix = 32 × 32, FOV = 210 × 210 mm) covering the whole brain.

### Data Analysis

First, we deleted the first 10 time points of the functional images because of the possible instability of the initial MRI signal and inadaptation to the scanning environment. The remaining data were conducted as standard for form transformation. The data preprocessing of the remaining data were done with the following steps, including the slice timing, head motion correction and spatial normalization to the Montreal Neurological Institute (MNI) space. The data of volunteers with >1.5 mm maximum translation or/and >1.5° degree of rotation in any directions were dislodged. After which, the remaining images were resampled at a resolution of 3 × 3 × 3 mm^3^ during the step of spatial normalization. Linear regression was applied to remove the effects of spurious covariates, including the Friston 24 head motion parameters, white matter signal and cerebrospinal fluid signal. Next, the functional images were entered into linearly detrended and temporal bandpass filter (0.01–0.1 Hz).

### Calculation of Long FCD and Shortfcd Calculation Maps

The local and global FCD maps for each individual were calculated in a gray matter (GM) mask. The number of functional connections of a given voxel was considered as a degree of a node in a binary graph. First, we defined the functional connectivity between a given voxel with each of the other voxels in the whole brain with a correlation threshold of *r* > 0.25 (17). Second, the binarized longFCD and shortFCD were defined based on the neighborhood strategy. We defined the voxels with a correlation threshold of *r* > 0.25 inside their neighborhood (radius sphere ≤ 6 mm) as shortFCD, and defined the voxels with a correlation threshold of *r* > 0.25 outside their neighborhood (radius sphere > 6 mm) as longFCD. Next, the shortFCD and longFCD maps of each subject were divided by the mean value so as to convert to Z scores to improve the normality. Finally, the shortFCD and longFCD maps underwent spatial smoothing with a Gaussian kernel of 6 × 6 × 6 mm^3^ full-width at half-maximum. The detailed procedure of the shortFCD and longFCD is given in a previous study (8).

In this study, we repeated the network analysis using a range of correlation r thresholds, to determine whether between-group differences were substantially affected by the selection of different *r*-value thresholds or nodes, used to construct brain networks (7). We used seven r thresholds (*r* = 0.30, 0.35, 0.40, 0.45, 0.50, 0.6, and 0.75). Here, we adopted the threshold of *r* = 0.45 to calculate the binarized FCD maps.

### Statistical Analysis

Data are presented as mean ± standard deviation. The demographic characteristics (age, AUDIT score and years of education) were analyzed with independent sample unpair *t*-tests. The statistical threshold was set at *p* < 0.05.

Before comparing the between-group differences in the FCD maps, we first used sample *t-*tests to construct within-group statistical maps of shortFCD and longFCD for patients with alcohol dependence and healthy subjects to identify the network distributions of each group (*p* < 0.001, false discovery rate (FDR) corrected). Then, independent sample unpair *t*-tests were utilized to evaluate the voxel-wise differences between patients with alcohol dependence and healthy subjects with nuisance covariates (age and years of education) of no interest. The AlphaSim correction (threshold of individual voxel of *p* < 0.01, cluster level of *p* < 0.05 with contiguous voxel volume ≥ 1,080 mm^3^) was used to determine the statistical differences.

Pearson's correlation was used to evaluate the relationships between those binarized FCD differences in brain areas and demographic characteristics. The statistical threshold was set at *p* < 0.05.

## Results

### Sample Characteristics

The behavioral characteristics of the alcohol dependent and the healthy subjects are presented in the Table 1. Patients with alcohol dependence did not significantly differ from the healthy subjects in mean age (*t* = 0.083, *p* = 0.934) and mean education (*t* = 1.466, *p* = 0.149). The mean AUDIT score was higher in patients with alcohol dependence than that of the healthy subjects (*t* = 18.2, *p* < 0.001). In the alcohol dependence group, the mean duration of drinking history was (mean ± std, 27.46 ± 10.89 years, 7 ~ 45 years), the mean SADQ score was (20.21 ± 7.09), and the mean daily alcohol consumption was (237.5 ± 115.39) ml.

**Table 1:** Characteristics of alcohol dependent and healthy subjects.

	**Alcohol dependent**	**Healthy subjects**	***t*-value**	***p*-value**
**DEMOGRAPHICS**				
Mean age, years	47.83 ± 6.93	47.67 ± 6.99	0.083	0.934
Education, years	9.67 ± 3.09	8.33 ± 3.21	1.466	0.149
Years of drink, years	27.46 ± 10.89	N/A	N/A	N/A
SADQ score	20.21 ± 7.09	N/A	N/A	N/A
AUDIT score	24.08 ± 5.69	2.63 ± 0.97	18.2	< 0.001
Daily alcohol consumption, ml	237.5 ± 115.39	N/A	N/A	N/A

*Data are mean ± standard deviation values. SADQ, Severity of alcohol dependence questionnaire; AUDIT, Alcohol use disorders identification test; N/A, Not applicable*.

### Binarized FCD Differences

#### One Sample *t*-Tests

**Figure 1:**
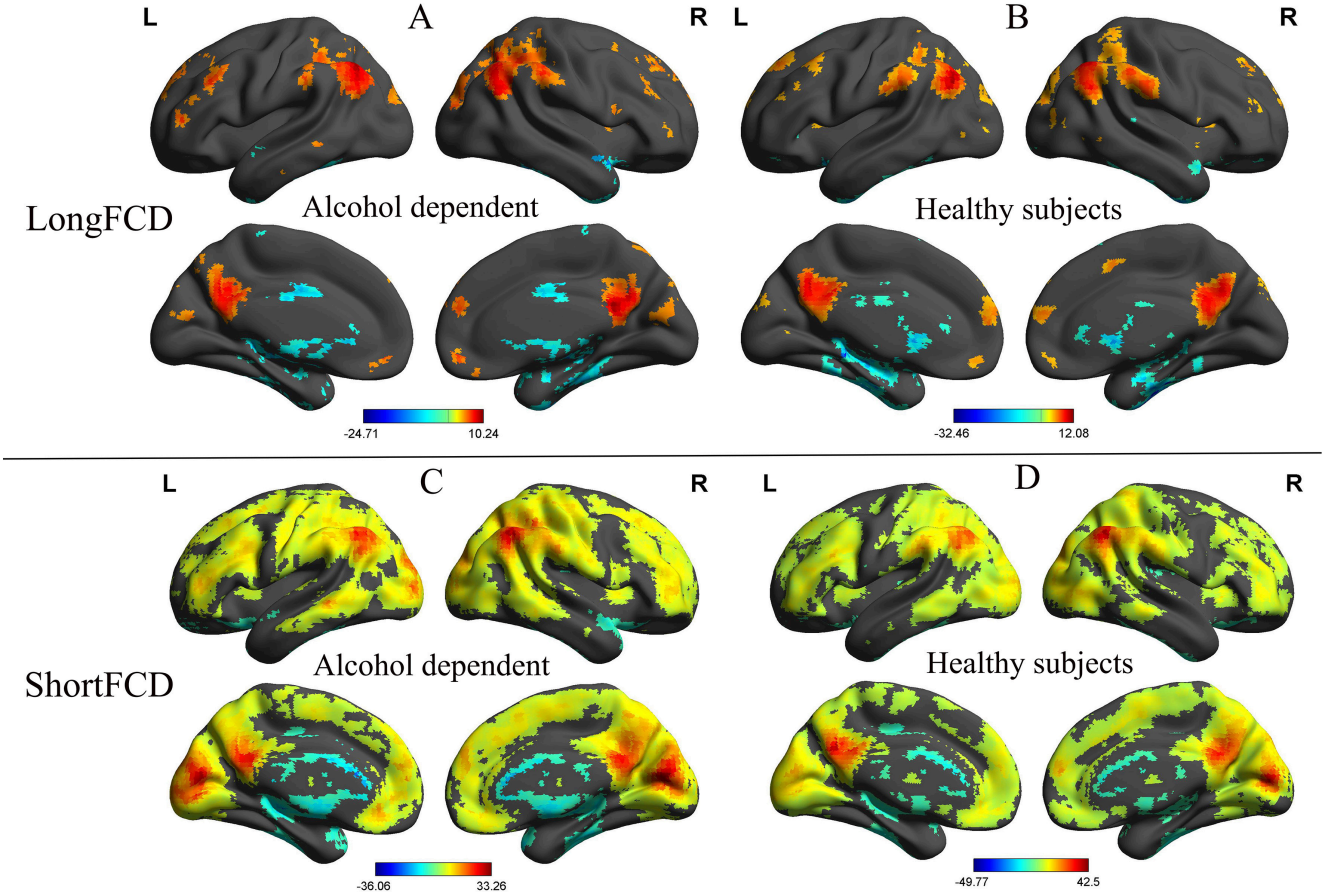
One sample *t*-test differences of alcohol dependent and healthy subjects in binarized FCD maps. The statistical threshold was set at FDR corrected voxel threshold of *p* < 0.001. Binarized longFCD **(A,B)** and shortFCD differences **(C,D)** in brain areas of patients with alcohol dependence and healthy subjects. FCD, Functional connectivity density; FDR, False discovery rate; R, right; L, left.

Before comparing the binarized FCD differences between the alcohol dependent and healthy subjects, one sample *t*-tests were used to construct within-group statistical maps for the alcohol dependence group and the healthy subjects, separately (*p* < 0.001, FDR corrected). Figure 1 shows the binarized longFCD and shortFCD maps in the alcohol dependence group and the healthy subject group (Figures 1A–D), respectively. We found that the two groups exhibited significantly similar differences in brain areas both in binarized longFCD and shortFCD maps. The covered differences of the brain areas in the binarized shortFCD were larger than the binarized longFCD for both groups. The covered differences of the brain areas in the binarized shortFCD in the alcohol dependent group were larger than that of the healthy subject group.

#### Two Sample *T*-tests

**Figure 2:**
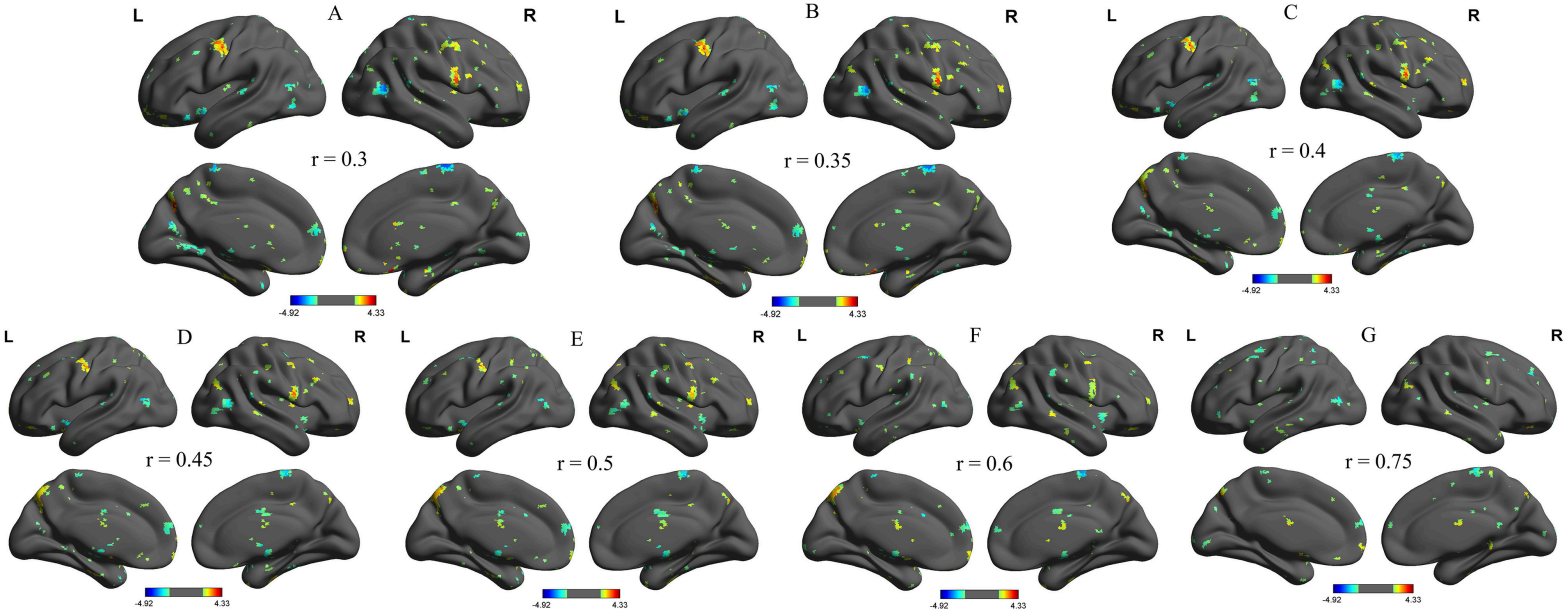
Binarized longFCD maps with several thresholds between alcohol dependent and healthy subjects. We analyzed binarized longFCD differences between alcohol dependent and healthy controls across seven different *r*-value thresholds (**A**, r = 0.30; **B**, 0.35; **C**, 0.40; **D**, 0.45; **E**, 0.50; **F**, 0.6; **G**, 0.75). Red color, increased binarized longFCD areas; Blue color, decreased binarized longFCD areas. R, right; L, left; longFCD, Long-range FCD.

**Figure 3:**
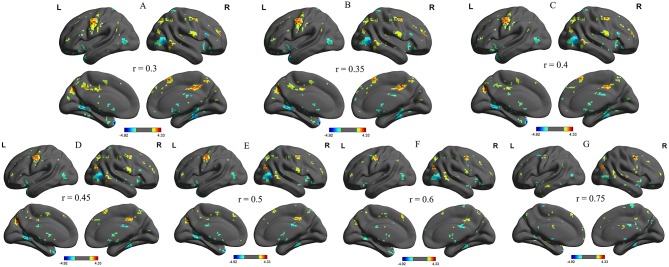
Binarized shortFCD maps with several thresholds between alcohol dependent and healthy subjects. We analyzed binarized shortFCD differences between alcohol dependent and healthy controls across seven different *r*-value thresholds (**A**, r = 0.30; **B**, 0.35; **C**, 0.40; **D**, 0.45; **E**, 0.50; **F**, 0.6; **G**, 0.75). Red color, increased binarized longFCD areas; Blue color, decreased binarized longFCD areas. R, right; L, left; shortFCD, Short-range FCD.

We further analyzed the binarized longFCD and shortFCD differences between the alcohol dependent group and the healthy controls, using several different *r*-value thresholds (*r* = 0.30, 0.35, 0.40, 0.45, 0.50, 0.6, and 0.75). We observed highly similar intra-group differences in the binarized longFCD and shortFCD maps when using these different thresholds (Figures 2, 3), indicating that the intra-group differences did not depend on the threshold used; therefore, we only reported the results of the binary network analysis using a threshold of *r* = 0.45 in which the covered differences in the brain areas were larger.

**Figure 4:**
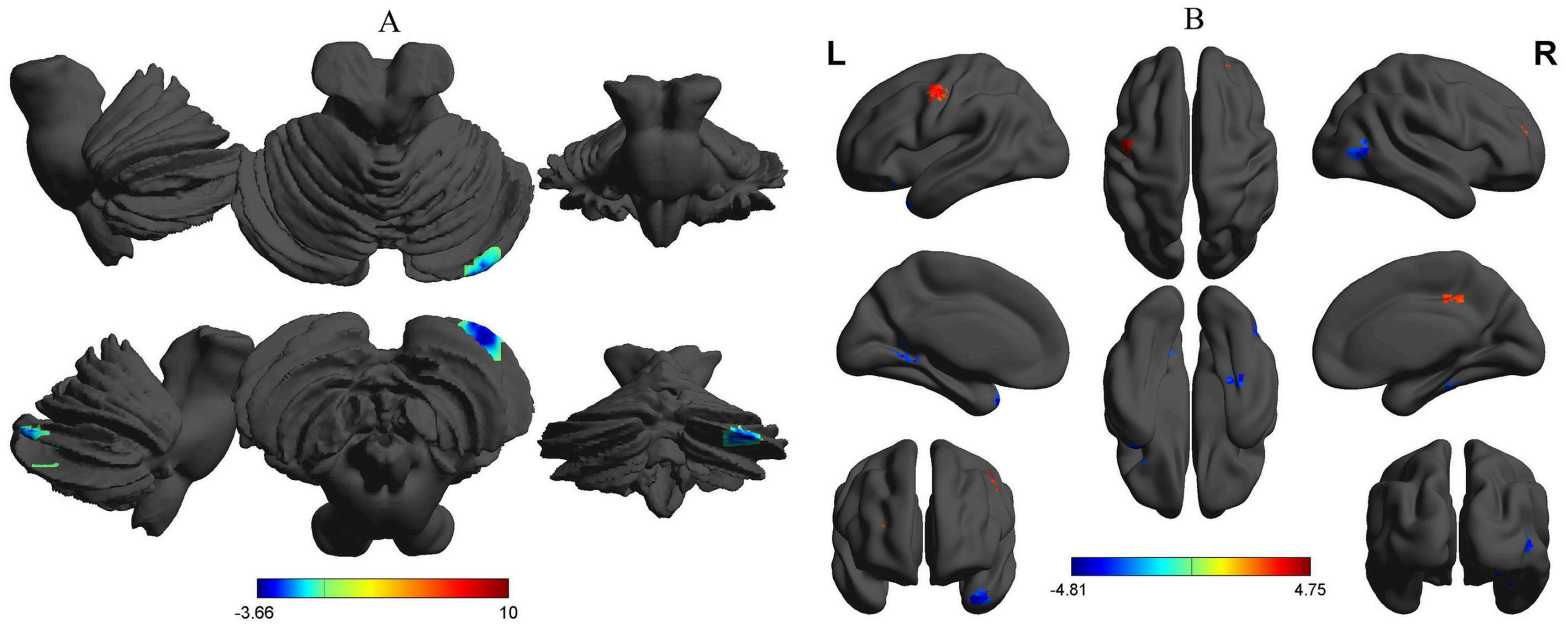
Binarized FCD differences between alcohol dependent and healthy subjects. Between-group differences in binarized longFCD **(A)** and shortFCD **(B)**. The statistical threshold was set at a corrected significance level of individual two-tailed voxel-wise *p* < 0.01 using an AlphaSim corrected threshold of cluster *p* < 0.05. FCD, Functional connectivity density; R, right; L, left; longFCD, Long-range FCD; shortFCD, Short-range FCD.

Compared with the healthy subjects, the alcohol dependent group showed a significant decreased binarized longFCD area in the right cerebellum posterior lobe (Table 2, Figure 4A). Compared with the healthy subjects, the alcohol dependent group showed significantly increased binarized shortFCD areas in the left precentral gyrus (BA 6), right medial frontal gyrus (BA 10, 46) in the salience network and the right cingulate gyrus (BA 31), and decreased binarized shortFCD areas in the left temporal pole (BA 38), bilateral visual association cortex (BA18, 19), left striatum cortex (lentiform nucleus) and the right inferior temporal lobe (fusiform gyrus extending to parahippocampal gyrus) (BA 20, 30, 37) (Table 2, Figure 4B).

**Table 2:** Binarized FCD differences between alcohol dependent and healthy subjects.

**Condition**	**Brain regions of peak coordinates**	**R/L**	**BA**	**Voxel volume (mm^**3**^)**	***t*-score of peak voxel**	**MNI coordinates**
						**X, Y, Z**
longFCD	Cerebellum posterior lobe	R	N/A	40	−3.6616	30, −75, −36
shortFCD	Superior temporal gyrus	L	38	83	−4.8138	−39, 21, −33
shortFCD	Fusiform gyrus, Parahippocampal gyrus	R	20, 30, 37	43	−3.4763	36, −33, −24
shortFCD	Lentiform nucleus	L	N/A	43	−3.6663	15, 12, −6
shortFCD	Lingual gyrus	L	18	41	−4.0418	−12, −48, −3
shortFCD	Middle occipital gyrus	R	19, 37	86	−4.0348	54, −69, 6
shortFCD	Medial frontal gyrus	R	46	51	4.3969	18, 48, 9
shortFCD	Precentral gyrus	L	6	74	4.7508	−45, −9, 45
shortFCD	Cingulate gyrus	R	31	40	3.6171	9, −30, 39

*The statistical threshold was set at a corrected significance level of an individual two-tailed voxel-wise p < 0.01 using an AlphaSim corrected threshold of cluster p < 0.05. Positive values of t-score indicate the increased longFCD or shortFCD in brain areas, and negative values of t-score indicate the decreased longFCD or shortFCD in brain areas. FCD, Functional connectivity density; shortFCD, Short-range FCD; longFCD, Long-range FCD; R, right; L, left; BA, Brodmann's area; MNI, Montréal neurological institute; N/A, Not applicable*.

#### ROC Curve

**Figure 5:**
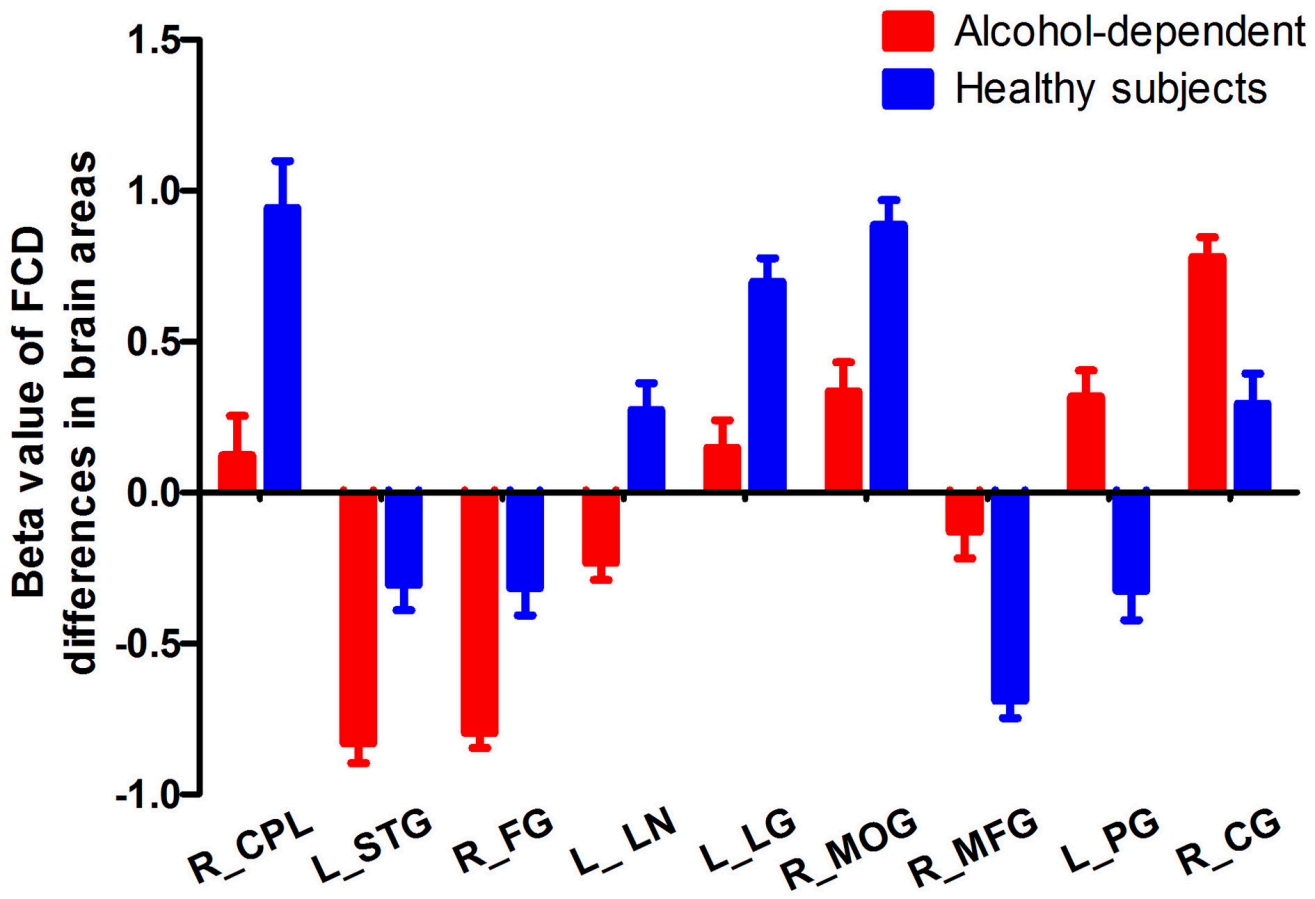
Mean beta value of FCD differences in regional brain areas. FCD, Functional connectivity density; R, right; L, left; CPL, Cerebellum posterior lobe; STG, Superior temporal gyrus; FG, Fusiform gyrus; LN, Lentiform nucleus; LG, Lingual gyrus; MOG, Middle occipital gyrus; MFG, Medial frontal gyrus; PG, Precentral gyrus; CG, Cingulate gyrus.

**Figure 6:**
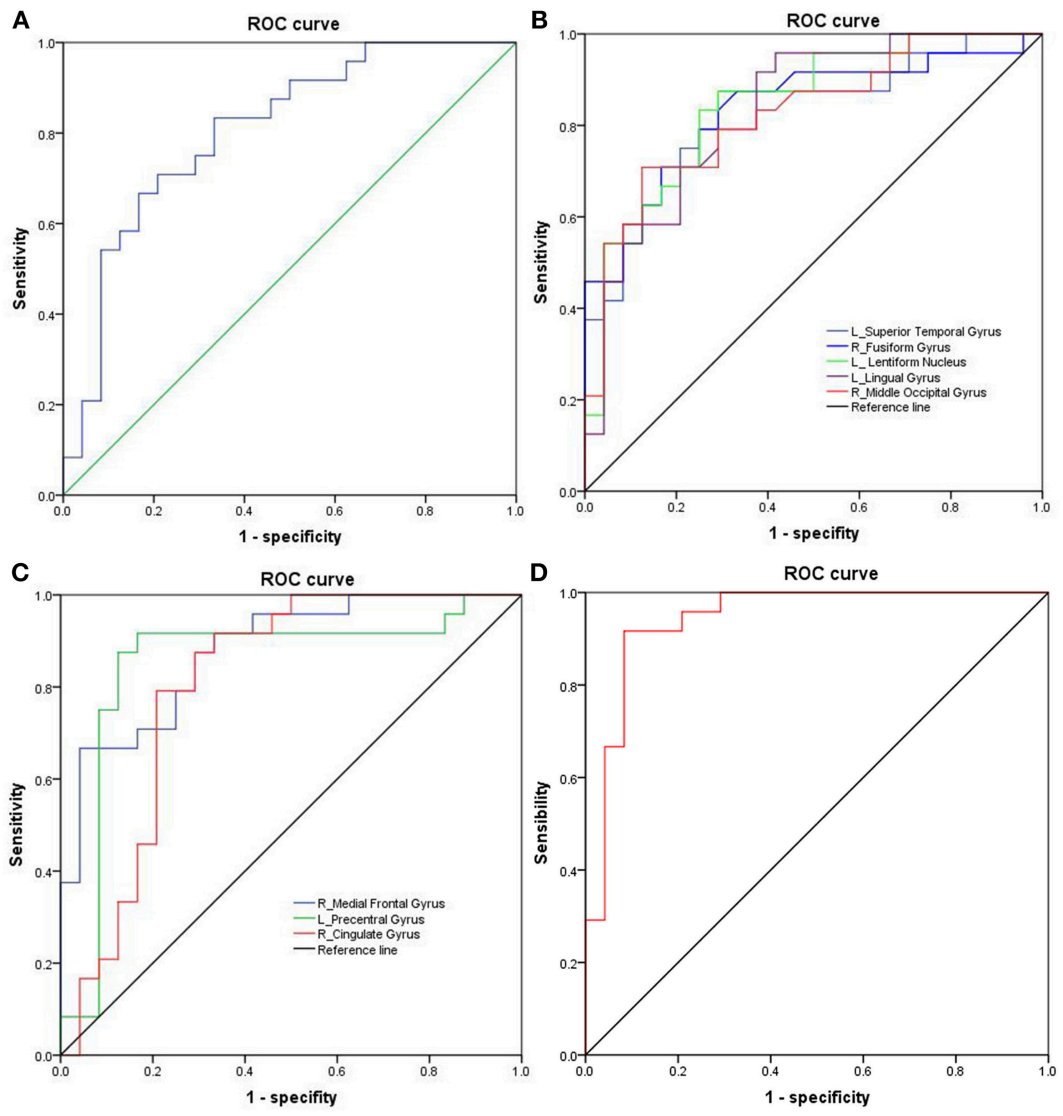
ROC curve analysis of FCD differences in regional brain areas. **(A)** Binarized longFCD difference in the right cerebellum posterior. **(B)** Decreased binarized shortFCD differences. **(C)** Increased binarized shortFCD differences. **(D)** Combined areas of visual pathway (right fusiform gyrus, left lingual gyrus, right middle occipital gyrus). ROC, Receiver operating characteristic; FCD, Functional connectivity density; R, right; L, left.

The mean beta value of the binarized shortFCD and longFCD differences in those altered brain areas were extracted (Figure 5). These binarized FCD differences in the brain areas were further used in a ROC curve, to evaluate the ability in distinguishing the alcohol dependent group from the healthy subjects (Figures 6A–C). The area under the curve (AUC) value, of the binarized longFCD difference in the right cerebellum posterior lobe was 0.806. Furthermore, the ROC curve demonstrated that this area alone discriminated the alcohol dependent group from the healthy subjects, with a sensitivity of 70.8% and a specificity of 79.2% (Table 3, Figure 6A). The AUC values, of the binarized shortFCD differences in brain areas were (0.838 ± 0.021; 0.806~0.878). Furthermore, the ROC curve demonstrated that the binarized shortFCD differences in those regional brain areas alone discriminated between the two groups with a high degree of sensitivity (81.26 ± 9.19%; 66.7~91.7%) and specificity (78.64 ± 10.31%; 62.5~95.8%) (Table 3, Figures 6B,C). The AUC value, of the combined areas of visual pathway that discriminated the alcohol dependent group from the healthy subjects, was up to 0.943, accordingly, the sensitivity and specificity were up to 91.7 and 91.7% with a cut-off point of 0.4629 (Table 3, Figure 6D).

**Table 3:** ROC curve for FCD differences in brain areas between alcohol dependent and healthy subjects.

**Brain area**	**AUC**	**Sensitivity, %**	**Specificity, %**	Cut off Point^*^
R_Cerebellum Posterior Lobe	0.806	70.8	79.2	0.353
L_Superior Temporal Gyrus	0.83	87.5	70.8	−0.798
R_Fusiform Gyrus	0.833	79.2	75	−0.6405
L_ Lentiform Nucleus	0.844	83.3	75	−0.0515
L_Lingual Gyrus	0.832	91.7	62.5	0.2195
R_Middle Occipital Gyrus	0.826	70.8	87.5	0.84
R_Medial Frontal Gyrus	0.878	66.7	95.8	−0.277
L_Precentral Gyrus	0.851	91.7	83.3	−0.173
R_Cingulate Gyrus	0.806	79.2	79.2	0.5365
R_Fusiform Gyrus + L_Lingual Gyrus+R_Middle Occipital Gyrus	0.943	91.7	91.7	0.4629

* *Cut-off point of mean FCD signal value. ROC, Receiver operating characteristic; FCD, Functional connectivity density; AUC, Area under the curve; R, Right; L, Left*.

### Pearson's Correlation Analysis

**Figure 7:**
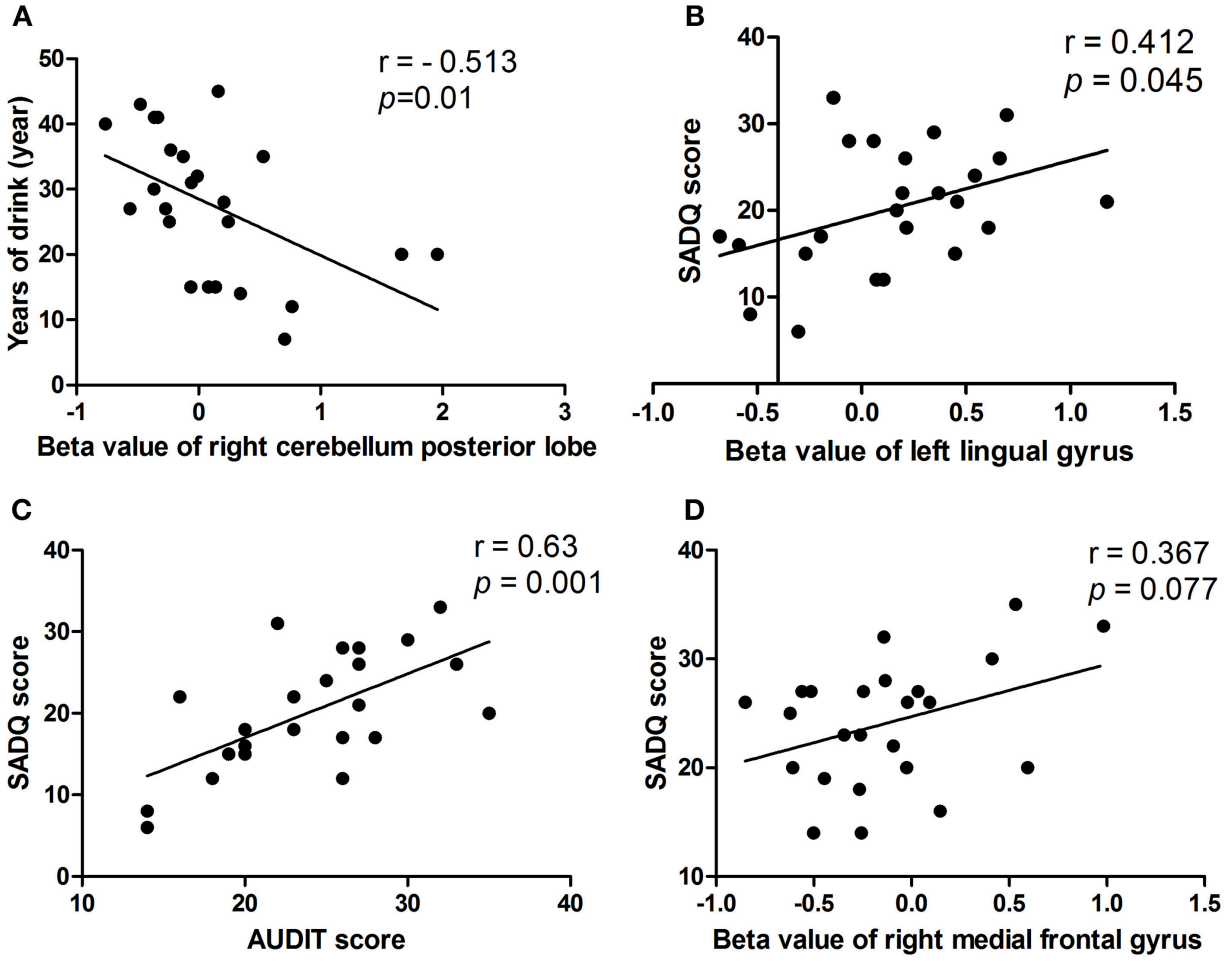
Pearson's correlation among characteristics of alcohol dependent and beta value of FCD differences in brain areas. In the alcohol dependent group, mean years of drinking negatively correlated with right cerebellum posterior lobe **(A)**. SADQ correlated with left lingual gyrus **(B)**, AUDIT score **(C)**, and right medial frontal gyrus **(D)**. FCD, Functional connectivity density.

In the alcohol dependent group, the mean years of drinking displayed a negative correlation with the beta value of the decreased binarized longFCD in the right cerebellum posterior lobe (*r* = −0.513, *p* = 0.01; Figure 7A). SADQ showed positive correlations with the beta value of the binarized shortFCD in the left visual association cortex (*r* = 0.412, *p* = 0.045; Figure 7B) and AUDIT score (*r* = 0.63, *p* = 0.001; Figure 7C), and an approximate positive correlation with the beta value of the increased binarized shortFCD area in the right medial frontal gyrus (*r* = 0.367, *p* = 0.077; Figure 7D). Other significant correlations between the beta value of brain areas with FCD differences and clinical features were not found (*p* > 0.05).

## Discussion

The current study is the first to utilize the shortFCD and longFCD analysis to identify alcohol-related intrinsic functional connectivity patterns in 24 patients with an alcohol dependence relative to 24 status-matched healthy subjects, and their correlations with demographic characteristics. Female subjects were excluded to remove the influence of a gender factor (18, 19). A ROC curve was applied to identify the ability of those FCD differences in distinguishing between the two groups. Three results were revealed: (1) the covered differences in the brain areas in the binarized shortFCD were larger than the binarized longFCD for both groups. (2) the intra-group FCD differences did not depend on the selection of different *r*-value thresholds used. Alcohol dependence in patients was associated with the right cerebellum posterior lobe with significant decreased binarized longFCD, as well as the left precentral gyrus, right salience network and right cingulate gyrus with increased binarized shortFCD, and the left temporal pole, right inferior temporal lobe (BA 20, 37), bilateral visual association cortex and left striatum cortex with decreased binarized shortFCD. (3) Recently, the ROC curve was widely applied in the exploration of the reliability of one neuroimaging approach, as a potential indicator in distinguishing one group from the other group (4, 19, 20). In general, an AUC value between 0.9 and 1 is considered excellent, and a value between 0.8 and 0.9 is considered good. In the present study, the ROC curve revealed good AUC values for those specific brain areas, and further diagnostic analysis demonstrated that those specific brain areas alone discriminated between patients with alcohol dependence and healthy subjects, with a high degree of sensitivity and specificity; (4) In the alcohol dependent group, the cerebellum posterior lobe, visual association cortex and salience network displayed significant correlations with demographic characteristics.

The visual pathway is divided into an object and spatial properties processing pathway (21–23). The object properties processing pathway runs from the striatum cortex down to the occipital lobe and inferior temporal lobe (BA 20, 37), and has been called the ventral system; this system processes properties of objects, such as shape, face, color and size. A decreased FCD value in the functional hubs may reflect less correlated activity and impairment of these functional hubs in facilitating network communications. In alcoholics, the concept of inefficiency includes difficulties in isolating irrelevant information (24), which is necessary for discriminating the targets from the distractors (6). In the present study we found that alcohol dependence was associated with decreased shortFCD areas in the ventral system, which showed a positive correlation with the SADQ score. Furthermore, the combined areas of visual pathway alone discriminated the alcohol dependent group from the healthy subjects, with a sensitivity of 91.7% and a specificity of 91.7%. These findings may reflect disorganization of the visual pathway caused by extravagant alcohol consumption, leading to inefficiency in information transmission from one place to another in the ventral system.

Morphology-anatomical studies have found decreased gray matter volumes in the cerebellum (25, 26) in patients with an alcohol dependence. These changes have shown to be predictive of the relapse risk, suggesting a significant role of decreased gray matter volumes in the cerebellum in clinical outcomes in alcohol dependence (27). Similarly, resting-state functional connectivity studies also showed consistent findings of decreased functional connectivity in the cerebellum (28–30). The cerebellum posterior lobe is associated with the regulation of coordinating movement, and was particularly vulnerable to alcoholism-related damage (18, 19, 31). The cerebellar circuit has been associated with motor control function and motor behavior, which are disrupted by alcohol intoxication (6, 32). Poor regulation of coordinating movement, balance and emotional changes are core characteristics of alcohol dependence (31, 33). Our results revealed that alcohol dependence was associated with a decreased longFCD in the cerebellum posterior lobe, which has shown altered functional connectivity in several studies in patients with alcohol dependence (6, 32–35), and may therefore support our results. Furthermore, the cerebellum posterior lobe was significantly correlated with the number of years of alcohol consumption. Therefore, we speculated that the decreased functional connectivity in the cerebellum posterior lobe may be interpreted as a functional impairment caused by long-term alcoholism, which may be one of the main reasons for impaired driving behavior in alcoholics.

In the present study we found significantly increased shortFCD in several brain areas. There are two prevalent speculations (6). One explanation of this finding could be interpreted as brain compensation, in which the alcoholic brain utilizes additional resources to help achieve the same level of performance as before. Long-term noxious effects of alcohol consumption on the human brain structure, both in gray and white matter, have widely been studied (36, 37). Another explanation of this finding could be interpreted as an enhanced neural effort to offset the noxious effects of alcohol consumption on the human brain structure.

## Conclusions

In summary, the longFCD and shortFCD analysis might serve as a biological indicator to observe the underlying intrinsic functional connectome changes in patients with alcohol dependence, with a high degree of discriminating ability. Specifically, the shortFCD analysis is more sensitive than the longFCD analysis in finding the differences in brain areas. In the present study, we found that the ventral visual pathway-cerebellar circuit deficit appears to be altered in patients with alcohol dependence, showing that alcohol dependence was associated with the longFCD deficit in the cerebellum posterior lobe, and the shortFCD deficit in the ventral system of the visual pathway. Our data highlights the role of functional connectivity and provides a new insight to better understand the neurobiological mechanisms that underlie alcohol dependence. These findings may help clinicians develop targeted intervention and prevention strategies. However, the relatively small sample size limited the significance. Future studies with a larger sample size are necessary to corroborate our findings.

## Author Contributions

LC and B-XL wrote the main manuscript text. LC, JZ, and X-JD conceived and designed the whole experiment. B-XL, RL, and X-JD collected the data. JZ and X-JD analyzed the data. X-JD revised the manuscript.

### Conflict of Interest Statement

The authors declare that the research was conducted in the absence of any commercial or financial relationships that could be construed as a potential conflict of interest.

## References

[1] Wise RA. Brain reward circuitry: insights from incensed incentives. Neuron (2002) 36:229–40. 10.1016/S0896-6273(02)00965-0 12383779

[2] Baker TB Piper ME McCarthy DE Majeskie MR Fiore MC. Addiction motivation reformulated: an affective processing model of negative reinforcement. Psychol Rev. (2004) 111:33–51. 10.1037/0033-295X.111.1.33 14756584

[3] Volkow ND Fowler JS Wang GJ. The addicted human brain: insights from imaging studies. J Clin Invest. (2003) 111:1444–51. 10.1172/JCI18533 12750391 PMC155054

[4] Dai XJ Liu CL Zhou RL Gong HH Wu B Gao L. Long-term total sleep deprivation decreases the default spontaneous activity and connectivity pattern in healthy male subjects: a resting-state fMRI study. Neuropsychiatr Dis Treat. (2015) 11:761–72. 10.2147/NDT.S78335 25834451 PMC4372006

[5] Lee S Lee E Ku J Yoon KJ Namkoong K Jung YC. Disruption of orbitofronto-striatal functional connectivity underlies maladaptive persistent behaviors in alcohol-dependent patients. Psychiatry Invest. (2013) 10:266–72. 10.4306/pi.2013.10.3.266 24302950 PMC3843019

[6] Luo X Guo L Dai XJ Wang Q Zhu W Miao X. Abnormal intrinsic functional hubs in alcohol dependence: evidence from a voxelwise degree centrality analysis. Neuropsychiatr Dis Treat. (2017) 13:2011–20. 10.2147/NDT.S142742 28814870 PMC5546828

[7] Liu X Zheng J Liu BX Dai XJ. Altered connection properties of important network hubs may be neural risk factors for individuals with primary insomnia. Sci Rep. (2018) 8:5891. 10.1038/s41598-018-23699-3 29651014 PMC5897381

[8] Tomasi D Volkow ND. Functional connectivity density mapping. Proc Natl Acad Sci USA. (2010) 107:9885–90. 10.1073/pnas.1001414107 20457896 PMC2906909

[9] Tomasi D Volkow ND Aging and functional brain networks. Mol Psychiatry (2012) 17:471:549–58. 10.1038/mp.2011.81 PMC3193908 21727896

[10] Everitt BJ Belin D Economidou D Pelloux Y Dalley JW Robbins TW. Review. Neural mechanisms underlying the vulnerability to develop compulsive drug-seeking habits and addiction. Philos Trans R Soc Lond Series B Biol Sci. (2008) 363:3125–35. 10.1098/rstb.2008.0089 18640910 PMC2607322

[11] Koob GF Volkow ND. Neurocircuitry of addiction. Neuropsychopharmacology (2010) 35:217–38. 10.1038/npp.2009.110 19710631 PMC2805560

[12] Tomasi D Volkow ND. Gender differences in brain functional connectivity density. Hum Brain Mapp. (2012) 33:849–60. 10.1002/hbm.21252 21425398 PMC3250567

[13] Tomasi D Volkow ND. Resting functional connectivity of language networks: characterization and reproducibility. Mol Psychiatry (2012) 17:841–54. 10.1038/mp.2011.177 22212597 PMC3323720

[14] Zhang Y Xie B Chen H Li M Liu F Chen H. Abnormal functional connectivity density in post-traumatic stress disorder. Brain Topogr. (2016) 29:405–11. 10.1007/s10548-016-0472-8 26830769

[15] Kong D Liu R Song L Zheng J Zhang J Chen W. Altered long- and short-range functional connectivity density in healthy subjects after sleep deprivations. Front Neurol. (2018) 9:546. 10.3389/fneur.2018.00546 30061857 PMC6054999

[16] Wang J Wei Q Yuan X Jiang X Xu J Zhou X. Local functional connectivity density is closely associated with the response of electroconvulsive therapy in major depressive disorder. J Affect Disord. (2018) 225:658–64. 10.1016/j.jad.2017.09.001 28910748

[17] Buckner RL Sepulcre J Talukdar T Krienen FM Liu H Hedden T. Cortical hubs revealed by intrinsic functional connectivity: mapping, assessment of stability, and relation to Alzheimer's disease. J Neurosci. (2009) 29:1860–73. 10.1523/JNEUROSCI.5062-08.2009 19211893 PMC2750039

[18] Dai XJ Gong HH Wang YX Zhou FQ Min YJ Zhao F. Gender differences in brain regional homogeneity of healthy subjects after normal sleep and after sleep deprivation: a resting-state fMRI study. Sleep Med. (2012) 13:720–7. 10.1016/j.sleep.2011.09.019 22503940

[19] Dai XJ Nie X Liu X Pei L Jiang J Peng DC. Gender differences in regional brain activity in patients with chronic primary insomnia: evidence from a resting-state fMRI study. J Clin Sleep Med. (2016) 12:363–74. 10.5664/jcsm.5586 26715399 PMC4773617

[20] Li HJ Dai XJ Gong HH Nie X Zhang W Peng DC. Aberrant spontaneous low-frequency brain activity in male patients with severe obstructive sleep apnea revealed by resting-state functional MRI. Neuropsychiatr Dis Treat. (2015) 11:207–14. 10.2147/NDT.S73730 25653530 PMC4311758

[21] Kosslyn SM Ganis G Thompson WL. Neural foundations of imagery. Nat Rev Neurosci. (2001) 2:635–42. 10.1038/35090055 11533731

[22] Kozhevnikov M Kosslyn S Shephard J. Spatial versus object visualizers: a new characterization of visual cognitive style. Memory Cogn. (2005) 33:710–26. 10.3758/BF03195337 16248335

[23] Blazhenkova O Kozhevnikov M The new object-spatial-verbal cognitive style model: theory and measurement. Appl Cogn Psychol. (2008) 23:638–63. 10.1002/acp.1473

[24] Nixon SJ Tivis R Ceballos N Varner JL Rohrbaugh J. Neurophysiological efficiency in male and female alcoholics. Prog Neuropsychopharmacol Biol Psychiatry (2002) 26:919–27. 10.1016/S0278-5846(02)00206-3 12369267

[25] Fein G Di Sclafani V Cardenas VA Goldmann H Tolou-Shams M Meyerhoff DJ. Cortical gray matter loss in treatment-naive alcohol dependent individuals. Alcohol Clin Exp Res. (2002) 26:558–64. 10.1111/j.1530-0277.2002.tb02574.x 11981133 PMC2435064

[26] Makris N Oscar-Berman M Jaffin SK Hodge SM Kennedy DN Caviness VS. Decreased volume of the brain reward system in alcoholism. Biol Psychiatry (2008) 64:192–202. 10.1016/j.biopsych.2008.01.018 18374900 PMC2572710

[27] Rando K Hong KI Bhagwagar Z Li CS Bergquist K Guarnaccia J. Association of frontal and posterior cortical gray matter volume with time to alcohol relapse: a prospective study. Am J Psychiatry (2011) 168:183–92. 10.1176/appi.ajp.2010.10020233 21078704 PMC3668974

[28] Habas C Kamdar N Nguyen D Prater K Beckmann CF Menon V. Distinct cerebellar contributions to intrinsic connectivity networks. J Neurosci. (2009) 29:8586–94. 10.1523/JNEUROSCI.1868-09.2009 19571149 PMC2742620

[29] O'Reilly JX Beckmann CF Tomassini V Ramnani N Johansen-Berg H. Distinct and overlapping functional zones in the cerebellum defined by resting state functional connectivity. Cereb Cortex (2010) 20:953–65. 10.1093/cercor/bhp157 19684249 PMC2837094

[30] Herting MM Fair D Nagel BJ. Altered fronto-cerebellar connectivity in alcohol-naive youth with a family history of alcoholism. Neuroimage (2011) 54:2582–9. 10.1016/j.neuroimage.2010.10.030 20970506 PMC3150517

[31] Sullivan EV Rosenbloom MJ Pfefferbaum A. Pattern of motor and cognitive deficits in detoxified alcoholic men. Alcohol Clin Exp Res. (2000) 24:611–21. 10.1111/j.1530-0277.2000.tb02032.x 10832902

[32] Rzepecki-Smith CI Meda SA Calhoun VD Stevens MC Jafri MJ Astur RS. Disruptions in functional network connectivity during alcohol intoxicated driving. Alcohol Clin Exp Res. (2010) 34:479–87. 10.1111/j.1530-0277.2009.01112.x 20028354 PMC2858246

[33] Tu X Wang J Liu X Zheng J. Aberrant regional brain activities in alcohol dependence: a functional magnetic resonance imaging study. Neuropsychiatr Dis Treat. (2018) 14:847–53. 10.2147/NDT.S158221 29606878 PMC5868577

[34] Zheng H Kong L Chen L Zhang H Zheng W. Acute effects of alcohol on the human brain: a resting-state FMRI study. Biomed Res Int. (2015) 2015:947529 10.1155/2015/947529 25705701 PMC4332461

[35] Wang L Chen Y Yao Y Pan Y Sun Y. Sleep deprivation disturbed regional brain activity in healthy subjects: evidence from a functional magnetic resonance-imaging study. Neuropsychiatr Dis Treat. (2016) 12:801–7. 10.2147/NDT.S99644 27110113 PMC4835129

[36] Durkee CA Sarlls JE Hommer DW Momenan R. White matter microstructure alterations: a study of alcoholics with and without post-traumatic stress disorder. PLoS ONE (2013) 8:e80952. 10.1371/journal.pone.0080952 24260518 PMC3832443

[37] Grodin EN Lin H Durkee CA Hommer DW Momenan R. Deficits in cortical, diencephalic and midbrain gray matter in alcoholism measured by VBM: effects of co-morbid substance abuse. Neuroimage Clin. (2013) 2:469–76. 10.1016/j.nicl.2013.03.013 24179800 PMC3777684

